# Nuts and bolts of the salt-inducible kinases (SIKs)

**DOI:** 10.1042/BCJ20200502

**Published:** 2021-04-16

**Authors:** Nicola J. Darling, Philip Cohen

**Affiliations:** MRC Protein Phosphorylation and Ubiquitylation Unit, University of Dundee, Dundee, U.K.

**Keywords:** AMPK-related kinase, CREB, CREB-regulated transcriptional co-activator (CRTC), histone deacetylase (HDAC), myocyte enhancer factor 2 (MEF2), salt-inducible kinase (SIK)

## Abstract

The salt-inducible kinases, SIK1, SIK2 and SIK3, most closely resemble the AMP-activated protein kinase (AMPK) and other AMPK-related kinases, and like these family members they require phosphorylation by LKB1 to be catalytically active. However, unlike other AMPK-related kinases they are phosphorylated by cyclic AMP-dependent protein kinase (PKA), which promotes their binding to 14-3-3 proteins and inactivation. The most well-established substrates of the SIKs are the CREB-regulated transcriptional co-activators (CRTCs), and the Class 2a histone deacetylases (HDAC4/5/7/9). Phosphorylation by SIKs promotes the translocation of CRTCs and Class 2a HDACs to the cytoplasm and their binding to 14-3-3s, preventing them from regulating their nuclear binding partners, the transcription factors CREB and MEF2. This process is reversed by PKA-dependent inactivation of the SIKs leading to dephosphorylation of CRTCs and Class 2a HDACs and their re-entry into the nucleus. Through the reversible regulation of these substrates and others that have not yet been identified, the SIKs regulate many physiological processes ranging from innate immunity, circadian rhythms and bone formation, to skin pigmentation and metabolism. This review summarises current knowledge of the SIKs and the evidence underpinning these findings, and discusses the therapeutic potential of SIK inhibitors for the treatment of disease.

## Introduction

The AMP-activated protein kinase (AMPK) is one of the most studied protein kinases, the huge interest in this enzyme stemming from the discovery that it is a key sensor of cellular energy charge and one of the intracellular targets of metformin (also called glucophage), the drug used most commonly to treat Type 2 diabetes [1–3]. For this reason, interest in AMPK has tended to overshadow the other members of the same subfamily, which are equally interesting and are collectively termed the AMPK-related kinases. Here, we begin to rectify this anomaly by reviewing current knowledge of the salt-inducible kinases (SIKs). The first member was identified over 20 years ago when rats fed on a high salt diet were found to induce a protein kinase in adrenal cortical tissue [4], initially termed SIK, but later SIK1 when two other protein kinases with closely related catalytic domains were identified, which are now called SIK2 (previously called QIK) and SIK3 (previously QSK) [5,6]. The mRNA encoding SIK1 is not only induced by a high dietary salt intake [4], but also by adrenocorticotropic hormone [4,7], circadian rhythms [8], glucagon signalling [9] and membrane depolarisation [10]. In contrast, SIK2 and SIK3 are expressed constitutively in many tissues and ligands that increase or decrease their level of expression have not been identified, although a recent paper reported that the expression of these isoforms is reduced in adipose tissue from insulin-resistant or obese individuals [11].

In this review, we provide an overview of current knowledge about the SIKs, their physiological roles and how they are regulated, highlighting the potential therapeutic benefits that SIK inhibitors may have for the treatment of a number of diseases.

## Evolution of the SIKs

There is only one gene encoding an SIK in the nematode *C.elegans,* termed KIN-29, which is an orthologue of mammalian SIK2 [12], whereas the fruit fly *Drosophila melanogaster* expresses both SIK2 and SIK3 [13], indicating that the genes encoding these SIK isoforms arose during invertebrate evolution. In contrast, all vertebrates from fish to mammals express all three SIK isoforms. Strikingly, the genes encoding *sik2* and *sik3* are located on the same chromosome in nearly all vertebrates whose genes have been sequenced. In mice, the genes encoding SIK2 and SIK3 are located on chromosome 9 and in humans on chromosome 11, where they are separated by only 4.6 Mb and 5.1 Mb, respectively. The gene encoding SIK1 is located on a separate chromosome, which is chromosome 17 in mice and chromosome 21 in humans. The reason for such close linkage of the genes encoding SIK2 and SIK3, which has been conserved through vertebrate evolution, is unclear but it may permit the transcription of these genes to be regulated co-ordinately. Interestingly, a recent duplication of the gene encoding SIK1 has occurred in humans creating an additional SIK family member, termed SIK1B, which like the gene encoding SIK1 is located on chromosome 21. The amino acid sequence of SIK1B is identical to that of SIK1, except for the replacement of Ala615 in SIK1 by Val in SIK1B.

## Phosphorylation and domain structure of the SIKs

### Activation of SIKs by LKB1-dependent phosphorylation

**Figure 1.**
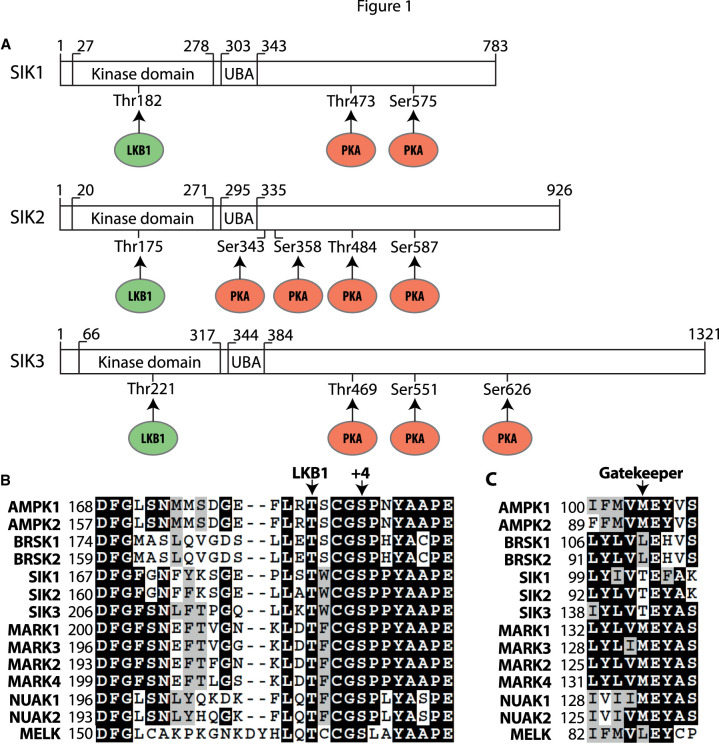
Domain structure of the SIKs and sites of regulatory phosphorylation. (**A**) Schematic of the domain structures of human SIK1 (Uniprot P57059), SIK2 (Q9H0K1) and SIK3 (Q9Y2K2) showing the threonine in the N-terminal kinase domain phosphorylated by LKB1 (green), the ubiquitin-associated (UBA) domain and the C-terminal tail with the sites of phosphorylation by PKA (red). The equivalent phosphorylation sites in murine SIK1 (Q60670) are Thr182, Thr475 and Ser577; the equivalent phosphorylation sites in murine SIK2 (Q8CFH6) are Thr175, Ser343, Ser358, Thr484 and Ser587 and the equivalent phosphorylation sites in murine SIK3 (Q6P4S6) are Thr163, Thr411, Ser493 and Ser616. (**B**) Alignment of the activation loop sequences of human AMPK-related kinases. Marked are the Thr phosphorylated by LKB1 and the conserved Ser at +4 reported to be a site of autophosphorylation. Identical residues are shaded in black and conserved residues are shaded in grey. (**C**) As in B except that the gatekeeper Thr residue is marked.

All three SIKs have an N-terminal protein kinase domain, followed by a ubiquitin-associated (UBA) domain and then a long C-terminal tail devoid of any recognisable structural feature (Figure 1A). Phosphorylation of a threonine residue in the activation loop of each SIK (Thr182 in SIK1, Thr175 in SIK2 and Thr221 in SIK3) is required for catalytic activity (Figure 1B). The mutation of these residues to Ala inactivates the SIKs [14] and has been used to generate knock-in mice expressing kinase-inactive mutants of each SIK isoform [15]. The protein kinase responsible for phosphorylating the activating threonine residues of SIKs *in vivo*, and indeed all the other 13 AMPK-related kinases, apart from MELK, is liver kinase B1 (LKB1) [14]. The critical role of LKB1 has been established by the absence of significant SIK activity in cells lacking LKB1 activity and expression, such as HeLa cells [14,16] or cells from LKB1 knock-out (KO) mice [14,17]. LKB1 is complexed with two other proteins in cells, MO25 and STRADα or STRADβ. The binding of STRAD and MO25 localises LKB1 to the cytosol and activates its kinase domain. This explains why LKB1 is constitutively active and hence why the SIKs are tonically active in cells. Although phosphorylation of the threonine residue in the activation loop of AMPK can be catalysed by calmodulin-dependent protein kinase kinase (CaMKK), and this mechanism operates to regulate AMPK in the central nervous system and in T cells [18], the SIKs are not activated in response to stimulation with a calcium ionophore or by overexpression of CaMKK2 in HeLa cells [19].

SIK1 and SIK2, but not SIK3, are reported to undergo autophosphorylation in mammalian cells at Ser186 and Ser179, respectively [20], which are located four amino acids C-terminal to the activating phospho-threonine residue (Figure 1B). This creates a consensus motif (TxxxS*, where x is any amino acid and S* is the phosphorylation site) for phosphorylation by glycogen synthase kinase 3 (GSK3) [21], which may permit GSK3 to phosphorylate Thr182 of SIK1 and Thr175 of SIK2, creating a positive feedback loop to ensure the activation of SIK1 and SIK2 is complete. This is an attractive hypothesis, but further experiments are needed to establish whether this occurs *in vivo*. In particular, it needs to be established whether Ser186 and Ser179 are phosphorylated to a significant stoichiometry in the endogenous SIK1 and SIK2 proteins, and whether SIK1/2 activity and Thr182/Thr175 phosphorylation are reduced in GSK3α and/or GSK3β KO cells, or in cells treated with specific GSK3 inhibitors, such as CHIR99021 [22]. It should also be noted that Ser186 and Ser179 of SIK1 and SIK2 are followed by a proline residue (Figure 1B), and do not conform to the preferred consensus sequence for phosphorylation by SIKs and other AMPK-related kinases, which is LxB(S/T)xS*xxxL (where B is a basic amino acid, x is any amino acid and S* is the site of phosphorylation) [5,23,24]. Ser186 and Ser179 might not therefore be sites of autophosphorylation but phosphorylated by one or more members of the CMGC subfamily of protein kinases that phosphorylate Ser/Thr-Pro sequences and which might have been trace contaminants in the SIK1 and SIK2 immunoprecipitates used to study ‘autophosphorylation’ in these experiments. This possibility should be investigated using one or more of the relatively specific and structurally unrelated SIK inhibitors that have now become available.

### Inhibition of the SIKs by cyclic AMP-dependent protein kinase

**Figure 2.**
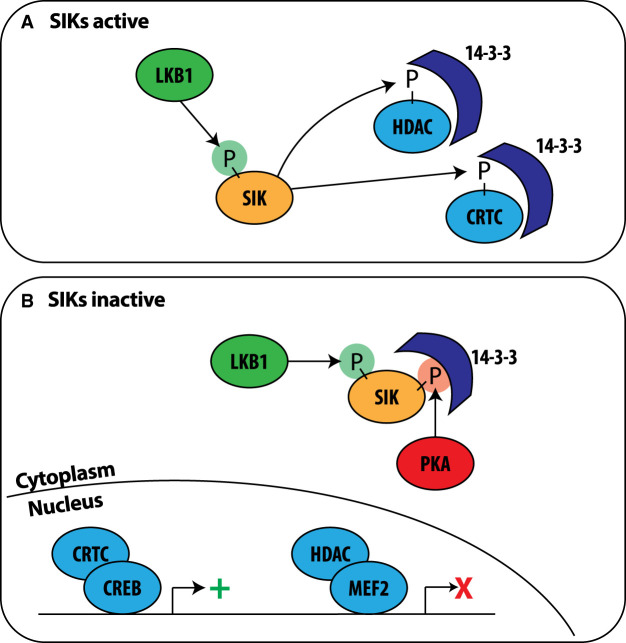
Consequences of the activation and inactivation of the SIKs by phosphorylation. (**A**) SIKs (orange) are activated by LKB1-dependent phosphorylation (green P) and phosphorylate (P) CRTC family members and Class 2a HDACs (HDAC4/5/7/9). These phosphorylated substrates (light blue) bind to 14-3-3s which retains them in the cytosol. (**B**) The SIKs are phosphorylated by PKA-dependent phosphorylation (red P) inducing binding to 14-3-3 proteins (dark blue) and inactivation. PKA-dependent phosphorylation may also promote SIK1 translocation from the nucleus to the cytoplasm (not shown). Inactivation of SIKs leads to the dephosphorylation and nuclear translocation of CRTCs and Class 2a HDACs. Within the nucleus CRTCs promote (green +) CREB-dependent gene transcription and Class 2a HDACs inhibit (red X) MEF2-dependent transcription.

In addition to the activating phosphorylation site(s) in the kinase catalytic domain, the C-terminal tails of the SIKs are phosphorylated by cyclic AMP-dependent protein kinase (PKA) (Figure 1A). Two serine residues in SIK1, four in SIK2 and three in SIK3 undergo phosphorylation by PKA *in vitro* [25], and in response to physiological or pharmacological stimuli that elevate intracellular levels of cyclic AMP (Table 1) [26–30]. These cyclic AMP-elevating stimuli induce the dephosphorylation of physiological substrates of the SIKs [28–31], indicating that phosphorylation by PKA inhibits SIK catalytic activity. However, a decrease in SIK activity induced by PKA-catalysed phosphorylation *in vitro* has not yet been demonstrated with any SIK isoform [27,28]. On the other hand, it has been shown that phosphorylation by PKA permits the SIKs to interact with 14-3-3 proteins (Figure 2). The phosphorylation of SIK1 at Thr473 and Ser575 drives the re-localisation of SIK1 to the cytosol and binding to 14-3-3 proteins [25,30,32]. In contrast, SIK2 and SIK3 are localised predominantly within the cytoplasm. PKA-dependent phosphorylation does not therefore induce the nucleo/cytoplasmic shuttling of SIK2 and SIK3 but, instead, phosphorylation of SIK2 at Ser343/Ser358 and Thr484/Ser587 generates two pairs of 14-3-3 binding sites [27,30] and the phosphorylation of SIK3 at Thr469 and Ser551 generates one 14-3-3 binding site [26,30]. It is therefore possible that the binding of 14-3-3s to the PKA-phosphorylated forms of SIKs is required for their inactivation. Evidence in support of this hypothesis was obtained in an experiment in which HEK293 cells were stimulated with forskolin, a cyclic AMP-elevating agonist, and the SIK2 bound to 14-3-3s was immunoprecipitated from the cell extracts and assayed with a peptide substrate. The activity of SIK2 was found to be partially reduced, compared with SIK2[Ser358Ala/Ser587Ala] which did not co-immunoprecipitate with 14-3-3 [30]. Although more definitive experiments still need to be performed, this would be analogous to the mechanism by which plant nitrate reductase is inactivated in the dark, the first functional role for 14-3-3s to be identified [33]. The LKB1-dependent phosphorylation of SIK1 and SIK3 has also been reported to create a 14-3-3 binding site [34] and may underlie the low level of 14-3-3 binding observed in cells not exposed to cyclic AMP-elevating agents; however there is a substantial increase in 14-3-3 binding to SIK1, SIK2 and SIK3 detected following PKA-dependent phosphorylation [26,27,30].

**Table 1:** Amino acid sequences surrounding the Ser/Thr residues in SIK1, SIK2 and SIK3 that are phosphorylated by PKA

SIK	Phosphorylation site	Sequence
SIK1	Thr473	STG**RRH** T* LAEVST
SIK1	Ser575	QEG**RR**A S* DTSLTQ
SIK2	Ser343	L**K**S**HR**S S* FPVEQR
SIK2	Ser358	G**R**Q**RR**P S* TIAEQT
SIK2	Thr484	SGQ**RRH** T* LSEVTN
SIK2	Ser587	**R**EG**RR**A S* DTSLTQ
SIK3	Thr469	LSM**RRH** T* VGVADP
SIK3	Ser551	PLG**RR**A S* DGGANI
SIK3	Ser626	SPV**RR**F S* DGAASI

S* and T* indicate the phosphorylated amino acid residues. Basic amino acid residues N-terminal to the sites of phosphorylation are highlighted in boldface type. All the phosphorylation sites lie in BBxS*/T* sequences that conform to the classical consensus sequence for phosphorylation by PKA (where B denotes any basic residue, most frequently Arg) [135].

In murine cortical neurons the expression of SIK2 was reported to be decreased during deprivation of oxygen and glucose and was blocked by KN93, an inhibitor of calmodulin-dependent protein kinases 1 and 4 (CaMK1, CaMK4). CaMK1 and CaMK4 were also reported to phosphorylate SIK2 at Thr484 when overexpressed in neurons [35]. Phosphorylation of SIK1 in kidney cells was blocked by KN93 and SIK1 was phosphorylated on Thr322 by CaMK1 *in vitro* [36]. Additional phosphorylation sites have been detected at the N-terminal end of the kinase domain and within the C-terminal tail of SIK2 and SIK3 in phospho-proteomic screens [37–40]. In summary, the SIKs may be regulated by phosphorylation at other sites by kinases distinct from PKA, LKB1 or the SIKs themselves, but more detailed investigation is needed to assess whether this is the case and the potential significance of these findings.

### The UBA domain of SIKs

Interestingly, ten AMPK-related kinases, including the SIKs, possess UBA domains and, indeed, they are the only protein kinases encoded in the human genome that possess this domain [41]. In other proteins UBA domains form ubiquitin or poly-ubiquitin binding motifs. However, despite conservation of the critical residues (Gly316, Leu326, Tyr338, Leu340 and Leu341 in SIK1) needed to form the hydrophobic interface required for ubiquitin binding, no interaction of Lys48-linked or Lys63-linked ubiquitin oligomers to either the UBA domains of SIK1, SIK2 or SIK3 or the full length proteins was detected [42]. Although binding to other ubiquitin species has not been reported, it currently seems that the main function of the UBA domain is to facilitate the LKB1-dependent phosphorylation of the activation loop threonine residue [42]. It is not yet clear how the UBA domain interacts with and alters the conformation of the kinase domain to allow phosphorylation by LKB1 to occur. A high resolution three-dimensional structure of an SIK isoform will be needed to understand this problem.

## Physiological substrates of the SIKs

Two important groups of substrates for the SIKs have been identified, namely the cyclic AMP-response-element binding protein (CREB)-regulated transcriptional co-activators (CRTC1, CRTC2 and CRTC3) and the Class 2a histone deacetylases (HDAC4, HDAC5, HDAC7 and HDAC9) (Table 2). Phosphorylation by SIKs induces the binding of CRTC family members to 14-3-3 proteins (Figure 2 and Table 2) in the cytosol from where they are unable to activate the transcription factor CREB in the nucleus. The inhibition of SIKs, either by PKA-dependent phosphorylation or pharmacological inhibition, allows the rapid dephosphorylation of CRTCs, their translocation to the nucleus and binding to CREB, thereby promoting CREB-dependent gene transcription (Figure 2) [17,23,43–46]. In addition, PKA can directly phosphorylate CREB at Ser133 to stimulate the transcription of CREB-dependent genes. However, CREB-dependent transcription of a subset of genes is still observed in cells in which endogenous CREB is replaced by the CREB[Ser133Ala] mutant [47]. It would therefore appear that PKA can activate CREB-dependent gene transcription via either or both of two mechanisms; phosphorylation and inhibition of SIKs and direct phosphorylation of CREB.

**Table 2:** Sites on CRTCs and Class 2a HDACs phosphorylated by the SIKs

Substrate	Phosphorylation site	Sequence	Reference
CRTC1	Ser151	SWRRTN S* DSALHQ	[16]
CRTC2	Ser171	ALNRTS S* DSALHT	[16,23]
CRTC2	Ser274	AMNTGG S* L**P**DLTN	[44]
CRTC3	Ser62	TQYHGG S* L**P**NVSQ	[17]
CRTC3	Ser162	ALNRTN S* DSALHT	[17,44]
CRTC3	Ser273	TLNTGG S* L**P**DLTN	[45]
CRTC3	Ser329	GLQSSR S* N**P**SIQA	[17]
CRTC3	Ser370	LRLFSL S* N**P**SLST	[17]
HDAC4	Ser246	PLRKTA S* E**P**NLKL	[31,44]
HDAC4	Ser467	PLGRTQ S* A**P**LPQN	[31]
HDAC5	Ser259	PLRKTA S* E**P**NLKV	[31]
HDAC5	Ser498	PLSRTQ S* S**P**LPQS	[31]

The Ser phosphorylated by SIKs lie in S*xP sequences (where S* indicates the phosphorylated amino acid residue), which is one of the consensus sequences for interaction with 14-3-3 binding proteins [136].

Phosphorylation of Class 2a HDACs by SIKs promotes 14-3-3 binding (Table 2) and the retention of HDACs in the cytosol. When SIKs are inactive, the Class 2a HDACs are dephosphorylated and translocate to the nucleus where they bind to myocyte enhancer factor 2 (MEF2) and repress MEF2-dependent gene transcription (Figure 2) [31,44,48,49]. It is currently thought that the Class 2a HDACs have little or no deacetylase activity but instead act as adaptors by recruiting Class 1 HDACs and other chromatin modifiers to transcription factor binding sites via interaction with MEF2 and other transcription factors [50,51]. Although phosphorylation by SIKs prevents the interaction of Class 2a HDACs with MEF2, it is unclear whether Class 2a HDACs are inactive in the cytosol or whether they have other cytosolic functions. For example, cytoplasmic HDAC4 is reported to promote vascular calcification through interaction with the cytoskeleton-associated protein ENIGMA in vascular smooth muscle cells [52].

## Development and exploitation of small molecule SIK inhibitors

**Figure 3.**
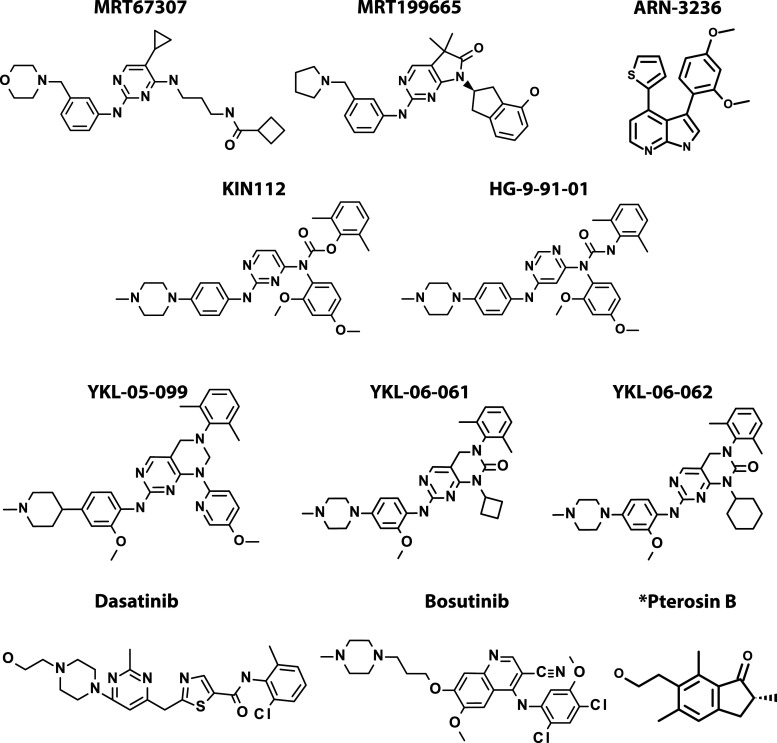
Chemical structures of compounds inhibiting SIKs. Chemical structures of compounds inhibiting SIKs at low nM concentrations that have been used to study the functions of SIKs in cells and *in vivo*. KIN-112, HG-9-91-01, YKL-05-099, YKL-06-061, YKL-06-062, Dasatinib and Bosutinib target the gatekeeper site on SIKs (Figure 1C) whereas MRT67307 and MRT199665 do not. *Pterosin B treatment mimics several effects of SIK3 KO in hepatocytes and chondrocytes but is not thought to be a direct inhibitor of SIK3.

A number of SIK inhibitors are now available and have been widely used to study the physiological roles of these protein kinases. It is therefore important to understand the caveats in the use of these reagents arising from their imperfect selectivity. The compound MRT67307 (Figure 3) was originally developed as a potent inhibitor of the IκB kinase (IKK)-related kinases TBK1 and IKKε [53]. Subsequently, it was found to enhance the secretion of the anti-inflammatory cytokine interleukin (IL)-10 and to suppress the production of pro-inflammatory cytokines in lipopolysaccharide (LPS)-stimulated monocytes and macrophages, which led to the discovery that these effects were mediated by inhibition of the SIKs [17]. The closely related compound MRT199665 (Figure 3) is an equally potent inhibitor of the SIKs, which does not inhibit TBK1 and IKKε, and is therefore an improved version of MRT67307 [17,54]. However, while MRT199665 is a relatively specific SIK inhibitor it also inhibits other AMPK-related kinases, and for this reason should be used in conjunction with HG-9-91-01 (Figure 3); the latter is a structurally unrelated SIK inhibitor that does not inhibit any other AMPK-related kinase family member [17].

The reason why HG-9-91-01 inhibits the SIK isoforms but does not inhibit any other AMPK-related kinases, is because SIKs are unique among these family members in possessing an amino acid with a small side chain (threonine) at the gatekeeper site (Figure 1C), which creates a small hydrophobic pocket near to the ATP-binding site that is targeted by HG-9-91-01. Importantly, the mutation of this threonine to an amino acid with a larger side chain generates SIK mutants with the same activity as the wild-type enzyme, but which are resistant to inhibition by HG-9-91-01 [17]. The inducible overexpression of such mutants has been exploited to investigate whether particular effects of HG-9-91-01 in cells are caused by SIK inhibition or by a non-specific ‘off-target' effect of this compound. We therefore recommend that two structurally unrelated SIK inhibitors be used routinely in cell-based studies, such as MRT199665 and HG-9-91-01, and wherever possible should also be used in combination with a drug-resistant SIK mutant or with SIK-deficient cells or cells expressing kinase-inactive mutants of SIKs. Such studies should identify physiological substrates and functions of the SIKs, more reliably than the deployment of just a single SIK inhibitor by itself. This should be born in mind when evaluating the sections that follow.

Although HG-9-91-01 is useful for studies of SIK function in isolated cells *in vitro*, its pharmacokinetic properties are unsuitable for *in vivo* studies. For this reason, YKL-05-099 (Figure 3) an analogue of HG-9-91-01 with superior pharmacokinetic characteristics has been developed to assess the *in vivo* roles of SIKs [55]. Two further compounds, YKL-06-061 and YKL-06-062 (Figure 3), have been developed to optimise their lipophicity and molecular size for topical use [56].

Like the SIKs, many protein tyrosine kinases contain a threonine residue at the gatekeeper site and most of the compounds developed as inhibitors of tyrosine kinases, including many drugs approved for the treatment of cancers (Cohen P., Cross D.R. and Jänne P. 2021 Nat Rev Drug Disc in press), target the hydrophobic pocket created by the gatekeeper threonine. Indeed HG-9-91-01 is a more soluble derivative of KIN112 (Compound 28 in [57]), which was originally developed as an inhibitor of lymphocyte cell-specific protein-tyrosine kinase (Lck), a member of the sarcoma kinase (Src) family of protein tyrosine kinases. HG-9-91-01 is also a potent inhibitor of Src family members and other tyrosine and serine/threonine kinases with threonine at the gatekeeper site [17]. Conversely, Dasatinib and Bosutinib, which are pan protein tyrosine kinase inhibitors approved for the treatment of chronic myelogenous leukaemia, are potent inhibitors of the SIKs, and SIK inhibition is likely to underlie the anti-inflammatory effects of Dasatinib [58].

In summary, the SIK inhibitors in current use have limitations and additional structurally unrelated SIK inhibitors with enhanced specificity are needed, including compounds selective for particular SIK isoforms. Compounds that may have improved specificity, such as ARN-3236 (Figure 3) are beginning to emerge now that interest in targeting SIKs for the treatment of disease is increasing (see SIK-inhibiting drugs for the treatment of disease).

## Physiological roles of the SIKs

### The role of SIKs in regulating macrophage function

The innate immune system is carefully balanced between a pro-inflammatory state, which is critical for the clearance of infection, and an anti-inflammatory state that is required to stabilise and protect tissues in the absence of infection and to aid tissue repair after infection. Macrophages are important because they have the potential to produce both pro-inflammatory and anti-inflammatory cytokines during pathogenic infection. A distinct profile of cytokines is secreted from macrophages depending on which pattern recognition receptor (PRR) is activated and what other inputs a macrophage receives. For example, bone marrow-derived macrophages (BMDM) can be induced to secrete high levels of the pro-inflammatory cytokines TNF and interleukin IL-12 if stimulated by bacterial LPS, but secrete high levels of the anti-inflammatory cytokine IL-10 in response to both LPS and cyclic AMP-elevating ligands, such as prostaglandin E2 (PGE2) [59,60]. The synthesis and secretion of PGE2 is itself driven by the LPS-stimulated induction of cyclooxygenase 2, the enzyme that catalyses the rate-limiting step in the synthesis of prostaglandins [61].

While ligation of Toll-Like Receptor 4 by LPS alone leads to activation of the transcription factors required for pro-inflammatory cytokine production, such as NF-κB and IRF5 [62], the combined stimulation with LPS and PGE2 is required for the production and secretion of high levels of the anti-inflammatory cytokine IL-10. Inhibition of the SIKs mediated by PKA-dependent phosphorylation (Figure 1A and Table 1) in response to PGE2, leads to the dephosphorylation of CRTC3 and its translocation to the nucleus, where it promotes CREB-dependent transcription of IL-10 in BMDM (Figure 2) [17,29], explaining why combined stimulation by LPS and PGE2 is needed for high levels of IL-10 production. In one report, the siRNA knock-down of CRTC3 in BMDM was sufficient to block PGE2 or SIK inhibitor-induced IL-10 expression, and the knock-down of CRTC1 and CRTC2 had no effect [17,29], while another laboratory reported that LPS and PGE2-stimulated IL-10 expression was suppressed in either CRTC3 KO or CRTC2 KO BMDM [63]. The reason for this discrepancy is unclear but might be related to differences in ligand concentrations used or the ways in which the macrophages were differentiated. Studies with macrophages from a variety of knock-in mice in which each SIK was replaced by a kinase-inactive mutant established that SIK2 and SIK3 activity is critical for the production of IL-10 in foetal liver-derived macrophages [15].

Incubation with ‘pan' SIK inhibitors suppressed the secretion of the pro-inflammatory cytokines TNF and IL-12 in BMDM [17], in bone marrow-derived dendritic cells [64] or human myeloid cells [65]. Similarly, LPS-stimulation of BMDM derived from knock-in mice expressing kinase-inactive mutants of each SIK isoform induced lower secretion of TNF, IL-12 and IL-6 than in wild-type BMDM. LPS-stimulated pro-inflammatory cytokine production was completely suppressed in foetal liver-derived macrophages from mice expressing kinase-inactive forms of both SIK2 and SIK3 [15]. The molecular mechanism by which SIKs stimulate the production of pro-inflammatory cytokines is unknown. However, it has been reported that SIK inhibition permits the dephosphorylation of HDAC4 and its translocation into the nucleus where it is proposed to bind to and promote the de-acetylation of the p65 subunit of NF-κB at Lys310, preventing NF-κB from binding to the *tnf* and *il12β* promoters [63]. Indeed, the suppression of LPS-stimulated secretion of pro-inflammatory cytokines by PGE2 was blocked in HDAC4 KO BMDM and the occupancy of p65 at the relevant promoters was restored [63]. The overexpression of the p65[Lys310Arg] mutant impaired NF-κB-dependent gene transcription in transfected HEK293T cells [66]. In summary, further research is required to determine whether HDAC4 directly de-acetylates p65, as de-acetylation of p65 has previously been attributed to Class 1 HDACs [67–69]. Further work is also needed to establish whether de-acetylation of p65 at Lys310 underlies the decrease in pro-inflammatory cytokine gene transcription detected in BMDM in response to SIK inhibition. The generation of a conditional knock-in mouse expressing the p65[Lys310Arg] mutant may be needed to establish this important point definitively.

### The role of SIKs in regulating mast cell function

Recently a role for SIKs in promoting the secretion of cytokines from mast cells has been identified. Like macrophages, mast cells are also innate immune cells, found in barrier tissues such as the gut, skin and lungs. Epithelial cells in these tissues release IL-33 in response to cell damage or necrosis, stimulating mast cells to release cytokines and chemokines. Treatment with two structurally unrelated pan-SIK inhibitors suppressed the secretion of multiple cytokines including IL-13, granulocyte-macrophage colony stimulating factor (GM-CSF) and TNF and chemokines including (C-C motif) ligand 2 (CCL2, also known as monocyte chemoattractant protein-1), CCL3 (macrophage inflammatory protein-1α), CCL4 (macrophage inflammatory protein-1β) and CCL24 (eotaxin-2). Secretion of IL-13, GM-CSF and TNF was partially suppressed in mast cells expressing the kinase-inactive SIK3[Thr163Ala] mutant or in SIK3 KO mast cells and was abolished in mast cells from SIK2[Thr175Ala]/SIK3[Thr163Ala] kinase-inactive double knock-in or SIK2/3 double KO mice. On the other hand, secretion of these cytokines was little affected in mast cells expressing SIK1[Thr182Ala]/SIK2[Thr175Ala] kinase-inactive double knock-in or in SIK2 KO mast cells. The lack of any effect on cytokine production in SIK2 KO mast cells may be explained by the greatly increased expression of SIK3 in these cells. The IL-33-dependent secretion of chemokines was also abolished in SIK2/3 double KO mast cells [54]. In summary SIK2 and SIK3 play a critical role in mediating the IL-33 stimulated secretion of cytokines and chemokines in mast cells, however the underlying molecular mechanisms have yet to be defined.

### The role of SIKs in regulating bone formation; implications for osteoarthritis and osteoporosis

Bone development and bone remodelling are both regulated by the peptide hormones, parathyroid hormone (PTH) and parathyroid hormone-related peptide (PTHrP), which also act to maintain mineral ion homeostasis. PTH and PTHrP are recognised by the PTH1 receptor, a transmembrane G protein-coupled receptor. As signalling via the PTH1 receptor promotes bone formation, there is considerable interest in manipulating this pathway in a therapeutic setting and indeed, PTH1 receptor agonists are currently used to treat osteoporosis [70]. Within bone, osteocytes express the highest copy numbers of the PTH1 receptor and are the most abundant cell type [71]. Stimulation of osteocytes in cell culture with PTH drives the activation of PKA and subsequent phosphorylation of SIK2, but apparently not SIK3 [72]. In osteocytes, activation of the PTH signalling pathway drives the CREB-dependent expression of receptor activator of NF-κB ligand (RANKL) [73] which acts on osteoblasts and osteocytes to promote bone resorption [74]. As anticipated, KO or knock-down of SIK2 in osteocytes allowed dephosphorylation and nuclear translocation of CRTC2 and increased CREB-dependent transcription of RANKL [72]. In addition to the production of RANKL, osteocytes also produce sclerostin via MEF2-dependent transcription [75], which negatively regulates bone formation [71]. When SIK2 activity is inhibited by PTH1 receptor-mediated PKA activation, this allows the dephosphorylation and nuclear translocation of HDAC4 and HDAC5, which inhibit MEF2 (Figure 2) to prevent the expression of sclerostin [72]. Through this mechanism, the inhibition of SIK2 in osteocytes promotes bone formation by tipping the balance of gene expression towards CREB-dependent rather than MEF2-dependent transcription. Using osteoblast and osteocyte-specific conditional KO models, the SIK2/3 double KO mouse was found to have increased bone mass and accelerated bone turnover, but this was not seen in SIK1, 2 or 3 single KO, SIK1/2 double KO or SIK1/3 double KO mice. This increase in bone mass was prevented by combining the HDAC4/5 double KO and SIK2/3 double KO in osteoblasts and osteocytes, indicating that HDAC4/5 may be key substrates driving the SIK2/3 KO phenotype [76]. Consistent with this finding, treatment of osteocytes *in vitro* with the pan-SIK inhibitor YKL-05-099 induced substantial changes in the gene expression profile which overlapped with that induced by PTH signalling, including up-regulation of RANKL and suppression of sclerostin expression [72]. Moreover, treatment of adult mice with YKL-05-099 promoted bone formation and increased bone mass [72], indicating the therapeutic potential of SIK inhibitors for the treatment of osteoporosis. Taken together, these studies establish that SIK inhibition is central to PTH1 receptor action in bone development and remodelling.

The importance of SIK3 in bone development was first highlighted by the small size and skeletal defects identified in SIK3 KO mice [77] and kinase-inactive SIK3[Thr163Ala] knock-in mice [15]. SIK3 KO mice displayed growth plate defects associated with a delay in chondrocyte differentiation (hypertrophy) and bone formation [77]. Moreover, human patients expressing SIK3[Arg129Cys], a mutation that reduces SIK3 kinase activity *in vitro*, have similar skeletal defects [78]. Although chondrocyte hypertrophy was delayed in mice with a chondrocyte-specific KO of SIK3 (cKO mice), SIK1 or SIK2 partially compensated for the absence of SIK3, as chondrocyte hypertrophy was delayed further in SIK1/3 or SIK2/3 double cKO mice [76]. Shorter proliferating chondrocyte regions were observed at birth when the *hdac4* gene was deleted in the SIK3 cKO mouse, supporting the idea that HDAC4 may act downstream of SIK3 to delay chondrocyte hypertrophy. In addition, the delayed chondrocyte hypertrophy observed in the SIK3 cKO mice was completely abrogated when the *hdac4* gene was deleted in the SIK3 cKO mice [76]. This suggests, but does not prove, that chondrocyte development is regulated by the SIK3-mediated phosphorylation of HDAC4 and its translocation to the cytosol. To establish whether this hypothesis is correct it will be necessary to also show that the delayed chondrocyte hypertrophy in SIK3 cKO mice is mimicked by replacing wild-type HDAC4 with the HDAC4[Ser246Ala/Ser467Ala] mutant in chondrocytes. This is because the SIK-catalysed phosphorylation of these residues appears to drive the nuclear exit of HDAC4 enabling MEF2 and/or Runt-related transcription factor 2-dependent gene transcription to occur (Figure 2 and Table 2) [31,79,80].

To further investigate the role of SIK3 in chondrocytes of adult mice, tamoxifen-induced SIK3 cKO mice were studied. These experiments showed an increase in both growth plate cartilage and articular cartilage which lubricates the ends of bones [81]. In osteoarthritis, articular cartilage chondrocytes undergo hypertrophy comparable to the terminal differentiation of growth plate chondrocytes [82]. As SIK3 deficiency delayed chondrocyte hypertrophy it was suggested that the KO or inhibition of SIK3 may protect against osteoarthritis. Indeed, there was thickening of the articular cartilage in adult SIK3 cKO mice due to increased chondrocyte proliferation and these mice were protected from developing osteoarthritis [81]. It has been reported that the intra-articular injection of mice with Pterosin B (Figure 3) protected against osteoarthritis [81] and based on experiments in primary chondrocytes the mechanism was suggested to involve the PKA-dependent inhibition of SIK3 followed by its proteasomal degradation [81,83]. It will clearly be of interest to investigate whether pan-SIK, or SIK3-specific inhibitors protect against osteoarthritis in mouse models of this disease.

### The role of SIKs in regulating circadian behaviour

Within the brain, important roles for the SIKs in regulating circadian rhythms and sleep have been uncovered. The suprachiasmatic nuclei (SCN) is the master regulator of physiological (24 h) cycles within the body, which co-ordinate sleep/wake timing, feeding and levels of activity, and regulate aspects of both physiology and behaviour. Circadian rhythms within cells are regulated by a series of negative feedback loops, for example the transcription factors CLOCK and brain/muscle ARNT-like protein 1 (BMAL1) are positive regulators of transcription, activated during the day, which drive the expression of the negative regulators Period (PER1, PER2 and PER3) and Cryptochrome. During the morning, BMAL1 and CLOCK levels increase, and drive the expression of PER, which accumulates during the evening and suppresses the activity of BMAL1 and CLOCK, blocking the continued transcription of PER and Cryptochrome. Meanwhile PER and Cryptochrome proteins are degraded, resetting the molecular clock overnight [84]. These circadian rhythms are adjusted in response to light exposure via the retina, re-aligning the system to changes in the light/dark cycle.

Both *sik1* and *per1* are targets of CREB-dependent gene transcription. However, as SIK1 prevents the activation of CREB by phosphorylating CRTC1 (Figure 2), this appears to be a feedback control mechanism for restricting the extent of CREB activation. Consistent with this notion, the siRNA-mediated knock-down of SIK1 in NIH3T3 cells allows the continued transcription and accumulation of PER1 [8], which mediates the adjustment of the circadian cycle to light [85]. When mice were exposed to light for 60 min during the dark phase, CREB-dependent gene transcription was activated inducing the transcription of *sik1* in the SCN. The siRNA knock-down of SIK1 in the SCN allowed an enhanced rate of adjustment to light when the light phase of the light/dark cycle was advanced, mimicking the effect of jet lag [8].

SIK3 KO mice also demonstrated abnormalities in circadian rhythm, but in contrast with SIK1 knock-down, this included a delay in adjustment to a shift in the light/dark cycle and a 6 h phase shift in circadian-regulated behaviours. The overexpression of SIK3, but not the kinase-inactive SIK3[Lys37Met] mutant, promoted phosphorylation and degradation of PER2 in NIH3T3 cells and, conversely, PER2 levels were elevated in liver and fibroblast cells from SIK3 KO mice. Thus the periodicity of circadian rhythms becomes dysregulated in SIK3 KO mice and cells from the SCN of these mice become rapidly desynchronised when they are cultured in the absence of additional inputs [86]. This is reminiscent of findings in Drosophila in which SIK3 was identified as a regulator of circadian behaviours in an RNAi screen, and SIK3 knock-down in Drosophila neurons disrupted circadian rhythms and the cycling of PER proteins in a non-cell-autonomous manner [87].

### The role of SIKs in sleep/wake regulation

Sleep/wake regulation occurs across many regions of the brain and not just the SCN. There are two components that drive sleep/wake cycles. First, sleep need which accumulates while an animal is awake and then dissipates during sleep; second, sleep latency or alertness, which is controlled by the activity of the circadian clock [88]. The introduction of random point mutations into mice identified a strain in which sleep time driven by increased sleep need was prolonged significantly. This increased sleep need was caused by a splice mutation resulting from a single nucleotide substitution in the splice donor site of intron 13 of the *sik3* gene, generating the SIK3^Slp/+^ mice in which exon 13 is deleted [89]. Comparison of the SIK3^Slp/+^ mice as a model of increased sleep need with a sleep deprivation model allowed the identification of 80 proteins in the brain whose phosphorylation increased with sleep need and decreased with a reduction in sleep need. A subset of these proteins in brain extracts co-precipitated preferentially with mutant SIK3 compared with wild-type SIK3 [90]. Intracerebroventricular injection of the pan-SIK inhibitor HG-9-91-01 reduced the phosphorylation of sleep-need-index-phosphoproteins (SNIPPs), and reduced sleep need in both sleep-deprived wild-type mice and SIK3^Slp/+^ mice as indicated by reduced slow-wave activity during non-rapid-eye movement sleep [90]. The *sik3^Slp^* mutation results in skipping of exon 13, which encodes a sequence of 52 amino acids in the C terminal tail of SIK3 and includes the PKA-dependent phosphorylation site Ser493 (equivalent to Ser551 in human SIK3) (Figure 1A and Table 1) [26,30,89]. Indeed, the Sleepy phenotype of increased sleep need was also seen in mice heterozygous for the SIK3[Ser493Ala] or the SIK3[Ser493Asp] mutation [91]. Loss of this inhibitory PKA-dependent phosphorylation site prevented 14-3-3 binding and inhibition of SIK3 [91] but did not increase intrinsic SIK3 kinase activity [90]. This is consistent with the notion that sustained SIK3 catalytic activity drives sleep need via phosphorylation of the SNIPPs. Loss of the PKA-dependent phosphorylation site in SIK1 (Ser577Ala, equivalent to Ser575 in humans) or SIK2 (Ser587Ala) also increased sleep need, but less markedly than in the SIK3 mutant mouse (Figure 1A and Table 1) [92].

Comparable effects have also been detected following SIK3 mutation in Drosophila [89] and following KIN-29 mutation in *C.elegans* [89,93]. The deletion of the catalytic domain of the SIK2 orthologue in *C.elegans* (KIN-29) (see Evolution of the SIKs) induced sleep defects [12]. However, *kin-29* deletion also decreased cellular ATP levels despite high adipose stores, indicating a failure to regulate metabolism. Whilst KIN-29 was required for sleep in satiated animals [94], sleep defects in *C.elegans* with *kin-29* deletion could be overcome by mobilising triglycerides from fat stores or by re-expressing *kin-29* in a subset of sensory neurons, which also corrected the fat phenotype [93]. Importantly, *kin-29 hda-4* double mutants had normalised sleep patterns and ATP levels [93]. Thus sleep defects in *C. elegans* caused by loss of SIK signalling correlated with energy deficits in the sensory neurons, indicating a close relationship between sleep need, detection of energy requirements and the response of fat-storage cells [93]. Further studies are needed to investigate if the regulation of sleep need by SIK3 occurs independently of metabolism in humans, and if the SIK-HDAC signalling pathway and alterations in metabolism are involved.

### The role of SIKs in depression

The SIKs have a role in the hippocampus in regulating depression. The underlying molecular mechanism of depression is not understood, but the activation of CREB, which drives the expression of brain-derived neurotrophic factor (BDNF) to promote neuronal survival and other growth factors that promote neurogenesis, is a core signalling pathway that is suppressed in mouse models of depression and up-regulated in response to anti-depressants [95,96]. Increased CREB activity in the hippocampus through CREB overexpression induced anti-depressant activity [97] and all major classes of anti-depressants increase CREB expression and activity in multiple regions of the brain including the hippocampus [95,98,99]. Hippocampal SIK2 has been reported to be up-regulated and CRTC1-CREB signalling suppressed in two different mouse models of depression (chronic social defeat stress (CSDS) and chronic unpredictable mild stress (CUMS)). Moreover, the overexpression of SIK2 in the hippocampus of mice induced depressive-like behaviour, while the knock-down or KO of SIK2 in the hippocampus prevented mice from displaying such behaviour when challenged with either the CUMS or CSDS models of depression [100]. Furthermore, the pan-SIK inhibitor ARN-3236, which crosses the blood-brain barrier when administered by intraperitoneal injection, induced anti-depressant effects in both models of depression [101]. Similar protection was observed when ARN-3236 was administered via stereotactic infusion into the hippocampus to reduce the possibility that SIK inhibition in the periphery mediates these effects [101]. Protection against CSDS or CUMS was associated with increased expression of BDNF driven by CRTC1-CREB dependent transcription [100,101].

### The role of SIKs in epilepsy

Genetic analysis of patients with developmental epilepsy identified six unrelated children with mutations in SIK1, but no previously identified genetic markers known to associate with epilepsy. Patients were identified with one of the following heterozygous SIK1 mutations; Pro287Thr, Ser411Cys, Gly636Ser or the truncation mutants Glu347X, Gln614X and Gln633X (where X indicates that the SIK1 sequence terminated at this residue). In all of the truncation mutants a stop codon was introduced C-terminal to the UBA domain in SIK1, leaving the UBA and kinase domains intact (Figure 1A). Indeed all of these SIK1 mutants retained their kinase activity as determined using *in vitro* kinase assays. On the other hand, the truncation mutants all displayed increased stability and increased cytoplasmic localisation compared with wild-type SIK1 [102]. As a result of multiple SIK1 mutations being identified in patients, a new subset of developmental epilepsy has been identified and termed SIK1 syndrome [103].

### The role of SIKs in regulating melanin production in the skin

The determination of skin pigmentation has two components, a genetically determined constitutive factor, and an adaptive component that produces changes in response to environmental stimuli. The presence of eumelanin (dark melanin) is a predictor of low skin cancer risk in response to UV irradiation [104] and therefore modulation of the adaptive component of skin pigmentation in the absence of harmful stimuli is potentially an attractive approach to protect against skin cancer. During UV-induced tanning, DNA damage induces the p53-mediated expression of proopiomelanocortin (POMC) in melanocytes and keratinocytes, which is then cleaved proteolytically to produce α-melanocyte stimulating hormone (α-MSH). The α-MSH is secreted and binds to the melanocortin 1 receptor (MC1R) on melanocytes. Ligation of the Gα_s_-coupled G protein receptor MC1R triggers the activation of adenylyl cyclase and elevation of the intracellular level of cyclic AMP, activating PKA. It was suggested that PKA phosphorylates CREB at Ser133 stimulating the transcription of CREB-dependent genes, such as microphthalmia-associated transcription factor (MITF), which drives melanogenesis [105]. In addition, PKA may inactivate one or more SIKs leading to the dephosphorylation and nuclear translocation of CRTCs (Figure 2), thereby activating CREB by a second mechanism. The evidence presented below indicates that the latter pathway may be the more significant. Elevation of cyclic AMP levels therefore results in the production of eumelanin, while at lower cyclic AMP levels it is mainly pheomelanin (yellow-red melanin) that is produced [105]. The human MC1R locus contains polymorphisms, with non-functional MC1R mutants associated with red-haired, fair-skin individuals, a poor tanning response and increased risk of developing skin cancers [104,106,107].

All three SIKs are expressed in melanocytes, but SIK2 appears to be the predominant isoform. In mouse B16 melanocytes siRNA-mediated knock-down of SIK2 induced the expression of CREB-dependent genes, including MITF, and melanogenesis in the absence of an extracellular stimulus [108]. This indicated that melanocytes may be primed for melanogenesis but this is restrained by the activity of SIK2. When melanocytes were exposed to UV irradiation, CRTC1 was rapidly translocated into the nucleus, promoting CREB-dependent gene transcription (Figure 2) and melanogenesis, an effect that was blocked by the overexpression of SIK2. When melanocytes were stimulated *in vitro* with forskolin, to activate PKA in the absence of UV irradiation, transcription of the MITF-dependent gene tyrosinase was blocked by the over-expression of wild type SIK2 or the SIK2[Ser587Ala] mutant (Figure 1A), but not by the kinase-inactive SIK2[Lys49Met] mutant [108]. Consistent with these findings, an increase in MITF and MITF-dependent gene transcription was also detected when melanocytes were treated *in vitro* with the pan-SIK inhibitor HG-9-91-01 [56].

Hair pigmentation in yellow-haired mice, which have an inactivating MC1R mutation, can be induced by subcutaneous injection of forskolin, which by-passes the requirement of α-MSH-MC1R signalling to increase intracellular cyclic AMP levels [108]. When the inactivating MC1R mutation was combined with SIK2 KO, these mice had brown (agouti-like) hair in the absence of forskolin injection [108]. While UV irradiation was unable to induce skin pigmentation in mice with the inactivating MC1R mutation, MC1R^e/e^, topical application of forskolin rescued eumelanin production and protected mice against UV-mediated cutaneous damage [109]. Similar results were obtained when the pan-SIK inhibitor HG-9-91-01 was applied topically to MC1R^e/e^ mice causing darker skin pigmentation without an additional stimulus, which was reversed when the treatment was stopped [56]. Moreover, the HG-9-91-01 derivatives YKL-06-061 and YKL-06-062 (Figure 3), which permeate human skin more efficiently, induced skin pigmentation when tested on human skin explants [56].

Melanin is produced in melanocytes, packaged into melanosomes and then transferred to keratinocytes [105]. Importantly, while SIK inhibitors increased the synthesis of eumelanin, they also stimulated the transfer of melanosomes into keratinocytes [56]. This raises the possibility that skin pigmentation could be induced by application of an SIK2 or a pan-SIK inhibitor in the absence of UV irradiation, and thus could be beneficial in protection against the skin damage and skin cancer induced by UV irradiation.

Topical treatment with forskolin also protected against UV-induced carcinogenesis when the MC1R^e/e^ mice were bred with UV-sensitive xeroderma-pigmentosum-complementation-group-C-deficient mice [109]. However, whether topical application of an SIK inhibitor would also protect these mice against UV-induced carcinogenesis, or whether there are additional benefits from forskolin treatment that are SIK-independent, has yet to be determined. The levels of SIK2 mRNA and protein were both decreased when mouse B16 melanocytes were subjected to UV irradiation [108], raising the possibility that this could be one of the mechanisms by which exposure to sun induces tanning.

### The role of SIKs in regulating metabolism

The coupling of feeding/fasting states to hepatic gluconeogenesis is regulated by the circulating levels of insulin and glucagon released by the pancreas. During prolonged fasting, glucagon up-regulates gluconeogenesis in hepatocytes, while in the fed state insulin inhibits the transcription of genes required for gluconeogenesis, such as those encoding glucose-6-phosphatase and phosphoenolpyruvate carboxykinase. Through the phosphorylation of CRTCs and Class 2a HDAC family members (Figure 2 and Table 2), SIKs keep the gluconeogenic programme repressed in the fed state [110], while insulin signalling via the activation of protein kinase B (PKB, also called AKT) represses the transcription of genes encoding gluconeogenic enzymes via the phosphorylation and inhibition of the FOXO family of transcription factors [111]. Under fasting conditions, the glucagon-stimulated increase in intracellular cyclic AMP activates PKA [112], which leads to the phosphorylation and inhibition of the SIKs (Figure 1A and Table 1) [30]. SIK inhibition in turn induces the dephosphorylation of CRTCs which translocate to the nucleus to promote CREB-dependent transcription (Figure 2) [9,28,113]. In parallel, Class 2a HDACs also undergo de-phosphorylation and translocation to the nucleus (Figure 2). In the nucleus HDAC4/5 are thought to recruit HDAC3 to deacetylate and activate FOXO family members, and together with CREB, permit transcription of the genes encoding gluconeogenic enzymes [114,115].

The initial genetic evidence of a role for AMPK-related kinase family members in gluconeogenesis came from studies in mice with a liver-specific KO of LKB1. These mice displayed increased fasting hyperglycaemia and increased translocation of CRTC2 to the nucleus. The fasting blood glucose levels in these mice could be reduced by the shRNA knock-down of CRTC2 [116]. Importantly, CRTC2 phosphorylation and blood glucose levels were unaffected in mice with a liver-specific KO of the genes encoding both AMPKα1 and AMPKα2 [117], indicating that an LKB1-dependent, but AMPK-independent, pathway had a critical role in regulating hepatic gluconeogenesis. When primary mouse hepatocytes were treated with the pan-SIK inhibitor HG-9-91-01 this compound induced expression of gluconeogenic genes and increased glucose production. These effects were due to inhibition of the SIKs, and not to an off-target effect of the inhibitor, since the overexpression of an SIK2 mutant resistant to HG-9-91-01 prevented the effects of this compound [28]. The SIKs have therefore been proposed as a therapeutic target for the treatment of hypoglycaemia, since their inhibition induces gluconeogenesis [28]. Conversely, the overexpression of either SIK1 or SIK2 in the livers of diabetic mice is sufficient to normalise blood glucose levels [9].

It is still unclear which SIK isoforms are critical for the regulation of gluconeogenesis. No effects on hepatic gluconeogenesis were detected in mice with a liver-specific KO of SIK1 or SIK2, but mice in which both SIK1 and SIK2 were ablated or replaced by kinase-inactive mutants were not studied [28,118]. Primary hepatocytes from SIK3 KO mice that had not been stimulated with glucagon displayed increased expression of gluconeogenic enzymes and more extensive dephosphorylation of CRTC2 than wild type hepatocytes, which was not observed in SIK1 KO or SIK2 KO hepatocytes [83]. This suggests an important role for SIK3 in repressing gluconeogenesis in the fed state. However, unexpectedly, mice in which the gene encoding SIK3 was ablated in all cells were hypoglycaemic [119]. However, these mice are born at sub-Mendelian frequencies and have an adverse phenotype [77,119]. It may therefore be better to study the role of SIKs in blood glucose homeostasis using the more specific SIK inhibitors that are becoming available in conjunction with liver-specific KO of SIK3.

It has also been reported that PKB catalyses the phosphorylation of SIK2 at Ser358 in hepatocytes and that this underlies the insulin-induced suppression of gluconeogenesis [113]. In contrast, another study reported that insulin induced the phosphorylation of SIK2 at Ser587 in brown adipocytes [120]. However, a further laboratory failed to detect any insulin-induced phosphorylation of SIK2 at Ser358 and instead found that Ser358 was only phosphorylated in hepatocytes in response to glucagon. Moreover, the liver-specific ablation of SIK2 had no effect on the insulin-mediated suppression of gluconeogenesis [28]. Other investigators also failed to detect any insulin-induced phosphorylation of SIK2 in 3T3L1 adipocytes, even though PKB was phosphorylated at a site that induces its activation. They also only observed phosphorylation of SIK2 at Ser358 in response to cyclic AMP elevation [27]. It is therefore unlikely that SIK2, or other SIKs, are regulated by insulin or PKB.

The SIKs have been proposed to regulate the insulin response in adipocytes by controlling glucose uptake and the transcription of lipogenic genes. Studies with human and rat adipocytes have mostly focused on SIK2, as it is the most abundant SIK isoform in adipocytes [5,121]. SIK2 KO mice displayed increased serum levels of triglycerides and adiponectin [122] and CRTC2, CRTC3 and HDAC4 were phosphorylated by SIK2 in rodent and human adipocytes (Table 2) [44]. SIK2 knock-down using siRNA or adipocytes from SIK2 KO mice reduced expression of the GLUT4 glucose transporter, whereas siRNA knock-down of CRTC2 or HDAC4 elevated GLUT4 expression in rodent adipocytes [44]. However, the inhibition of SIKs with HG-9-91-01 did not suppress GLUT4 expression in human adipocytes, although glucose uptake was reduced [11]. Therefore, more investigation is needed to establish how SIKs regulate glucose uptake and lipogenesis in adipocytes and which substrates mediate these effects [110].

### The role of SIKs in tumorigenesis

LKB1 is a tumour suppressor and many cancer cells lack LKB1 expression. Consequently, these cancer cells are also deficient in AMPK-related kinase activity, including all three SIKs. This raises the question of whether one or more SIK isoforms mediate some or all of the tumour suppressive effects of LKB1. In non-small cell lung cancer models driven by the KRAS[Gly12Asp] mutation, the tumour suppressive activity of LKB1 has been reported to be dependent on SIK1 and SIK3. In these studies, lentivirus was used to deliver Cre-recombinase to drive the expression of KRAS[Gly12Asp] whilst simultaneously delivering CRISPR guides to disrupt SIK1 and SIK3 expression, which accelerated tumour growth. Similar results were obtained using Cre-recombinase to simultaneously drive the expression of KRAS[Gly12Asp] and knock-out SIK1, alongside CRISPR-mediated SIK3 disruption. The tumour size and neutrophil infiltration in these models of SIK1/3 disruption were comparable to those seen when LKB1 was knocked out alongside the expression of KRAS[Gly12Asp] [123,124]. SIKs have also been reported to act as tumour suppressors in G-protein α_s_-driven pancreatic tumorigenesis, where oncogenic mutations in G-protein α_s_ elevate intracellular cyclic AMP, and cause sustained inhibition of SIKs, thereby modulating lipid metabolism and tumorigenesis [125]. However, SIK isoforms appear to have both tumour suppressive and oncogenic functions dependent on the tumour type, as discussed in detail elsewhere [126]. Notably, the SIK inhibitor YKL-05-099 attenuated disease progression in two acute myeloid leukaemia (AML) mouse models, which was attributed to on-target inhibition of SIK3 based on overexpression of the SIK3 gatekeeper mutant in AML cells (Figure 1C) [127]. Similarly, the SIK inhibitor ARN-3236 sensitised ovarian cancer cells to paclitaxel treatment in a xenograft model [128]. In summary, the potential of SIK inhibitors as anti-cancer agents is likely to be limited to particular types of cancer.

## SIK-inhibiting drugs for the treatment of disease

**Figure 4.**
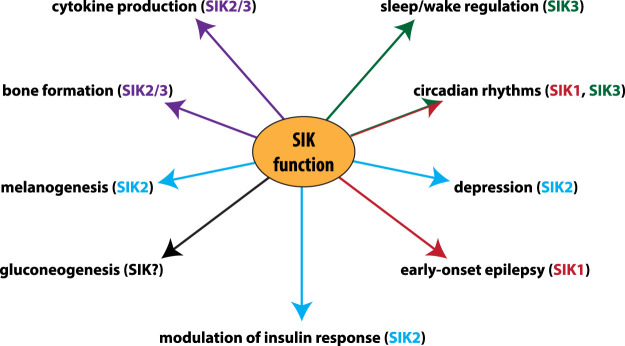
Physiological roles of different SIK isoforms identified by genetic analysis. The contribution of individual SIK isoforms to the regulation of physiological processes identified by the use of knock-out and/or knock-in mice. The major SIK isoform/s responsible for each role is colour coded; **SIK1** (red), **SIK2** (blue), **SIK3** (green), **SIK2 and SIK3** (purple). (**SIKs**) The SIKs have a role in modulating gluconeogenesis in mouse hepatocytes, but which SIK isoform(s) is responsible is unknown. In humans, mutations in SIK1 have been identified in patients with early-onset epilepsy. Consistent with the role in bone formation, a point mutation in SIK3 has been identified in a human patient with skeletal defects.

It should be apparent from the preceding sections that the SIKs not only have many physiological roles (Figure 4), but also that their inhibition could potentially be useful for the treatment of a number of diseases and conditions. One obvious area in which SIK inhibition may be beneficial is in the treatment of inflammatory diseases, since SIK inhibitors can switch macrophages from a pro-inflammatory to an anti-inflammatory state [17,64,65]. Indeed, the administration of anti-inflammatory macrophages to mice has been shown to attenuate LPS-induced endotoxic shock [129,130]. SIK inhibitors may also suppress other aberrant inflammatory responses driven by myeloid cells, such as those seen in rheumatoid arthritis, psoriatic arthritis and psoriasis, or in inflammatory bowel diseases where inflammatory myeloid cells are recruited to the gut and drive the production of inflammatory cytokines [55,131–133]. Since SIK inhibition suppresses the secretion of inflammatory cytokines and chemokines in mast cells [54], SIK-inhibiting drugs may also prove beneficial for the treatment of mast cell driven diseases, including asthma. However, as the normal function of cytokines is to co-ordinate the clearance of pathogens, the possibility that SIK-inhibiting drugs will increase the risk of microbial infection cannot be discounted. Therefore, as with other anti-inflammatory drugs, it will be important to assess whether the benefit outweighs the risk.

Apart from their potential to treat inflammatory diseases, SIK-inhibiting drugs may also have utility in the treatment of osteoporosis, due to the ability of SIK inhibitors to promote increases in bone mass by mimicking the effects of parathyroid hormone [72]. In addition, the topical application of SIK inhibitors could be beneficial in protection against skin damage and skin cancer induced by UV irradiation [56]. The discovery that two inhibitors of the Abelson tyrosine kinase, Dasatinib and Bosutinib, which have been approved for the treatment of chronic myelogenous leukaemia, are potent SIK inhibitors and that the anti-inflammatory properties of Dasatinib are likely to be attributable to SIK inhibition [58] has been encouraging, because it has provided reassurance that targeting the SIKs *in vivo* can be tolerated. For the above-mentioned reasons, it is not surprising that biotechnology companies have begun to focus on developing SIK-inhibiting drugs. On October 27th 2020, the pharmaceutical company Galapagos revealed that their Toledo programme to develop drugs to treat inflammatory and autoimmune diseases is focused on the development of SIK inhibitors (www.globenewswire.com/news-release/2020/10/27/2115341/0/en/Galapagos-R-D-Roundtable-showcases-Toledo-program.html). They announced that their dual SIK2/SIK3-selective inhibitor GLPG3970 had been well-tolerated in Phase I clinical trials and that it had entered Phase 2 for ulcerative colitis, rheumatoid arthritis and primary Sjögren syndrome, and Phase 1 trials for psoriasis and systemic lupus erythematosus. Another SIK inhibitor, GLPG4399, is currently in Phase 1 trials (clinicaltrials.gov). In addition, they mentioned that SIK inhibitors had shown promising pre-clinical activity in models of fibrotic disease, an area of unmet medical need. The biotechnology company, Soltego (www.soltego.com) is also focusing on developing SIK-inhibiting drugs but, in this case, on compounds that mimic the effects of sunlight and make the skin tan without exposure to the damaging effects of UV radiation. This may help to reduce skin cancer and even slow down the effects of ageing on the skin. The results of more advanced trials are awaited with interest.

## Outstanding questions

Although considerable progress has been made in recent years in understanding some of the physiological roles of the SIK isoforms and how they are regulated, several important outstanding questions have yet to be adequately addressed including the following:

1. Why are the genes encoding SIK2 and SIK3 situated so close to one another on the same chromosome and why has this been conserved throughout vertebrate evolution? Does this proximity permit co-ordinated transcriptional regulation of SIK2 and SIK3, or does it have some other function?
2. How are the SIK isoforms inhibited by PKA-dependent phosphorylation? Does their inhibition require the interaction of the phosphorylated proteins with 14-3-3s?
3. What are the roles of the long C-terminal domains of the three SIKs, which have no amino acid sequence similarities to one another and contain no recognisable structural domain? Will it be possible to crystallise the SIKs and solve their structures, to facilitate understanding of the functions of the C-terminal region and the UBA domain?
4. Currently, the CRTCs and the Class 2a HDACs are the only well-documented physiological substrates of the SIKs. Do many additional substrates remain to be discovered, which can explain why the SIKs are able to regulate the transcription of so many genes? For example, do the SIKs regulate other transcriptional co-activators and not just CRTCs? If SIKs prevent the deacetylation of transcription factors by driving the nuclear exit of Class 2a HDACs, do they also phosphorylate and activate the protein acetyl transferases that oppose HDAC action?
5. What are the identities of the protein phosphatases that dephosphorylate the threonine residue in the activation loops of SIKs (Figure 1B) that are required for activity? What are the phosphatases that reactivate the SIKs by dephosphorylating the sites targeted by PKA (Figure 1A), and which protein phosphatases dephosphorylate the substrates of the SIKs? Although SIK2 was reported to associate with protein phosphatase 2A (PP2A) [34,134] there are many PP2A complexes in which the catalytic subunits, PP2Aα and PP2Aβ, interact with different regulatory subunits that also direct subcellular location and determine substrate specificity. Which form(s) of PP2A interacts with each SIK isoform and what is the significance of the observation that the association of SIK2 with PP2A blocks the association of the latter with protein phosphatase methylesterase-1, an enzyme that methylates the C-terminal carboxylate of the PP2A catalytic subunits [134]?

Undoubtedly, these and other interesting questions about the SIKs and their regulation will provide fertile areas for future research.

## Abbreviations

AML: acute myeloid leukaemia
AMPK: AMP-activated protein kinase
BDNF: brain-derived neurotrophic factor
BMAL1: brain/muscle ARNT-like protein 1
BMDM: bone marrow-derived macrophages
CaMK: calmodulin-dependent protein kinase
CaMKK: calmodulin-dependent protein kinase kinase
CCL: (C-C motif) ligand
cKO: chondrocyte-specific knock-out
CREB: cyclic AMP-response-element binding protein
CRTC: CREB-regulated transcriptional co-activator
CSDS: chronic social defeat stress
CUMS: chronic unpredictable mild stress
GM-CSF: granulocyte-macrophage colony stimulating factor
GSK3: glycogen synthase kinase 3
HDAC: histone deacetylase
IKK: IκB kinase
IL: interleukin
KO: knock-out
Lck: lymphocyte cell-specific protein-tyrosine kinase
LKB1: liver kinase B1
LPS: lipopolysaccharide
MC1R: melanocortin 1 receptor
MEF2: myocyte enhancer factor 2
MITF: microphthalmia-associated transcription factor
MSH: melanocyte stimulating hormone
PER: Period
PGE2: prostaglandin E2
PKA: cyclic AMP-dependent protein kinase
PKB: protein kinase B
POMC: proopiomelanocortin
PP2A: protein phosphatase 2A
PRR: pattern recognition receptor
PTH: parathyroid hormone
PTHrP: parathyroid hormone-related peptide
RANKL: receptor activator of NF-κB ligand
SCN: suprachiasmatic nuclei
SIK: salt-inducible kinase
SNIPP: sleep-need-index-phosphoprotein
Src: sarcoma kinase
UBA: ubiquitin-associated
